# Mobile Aid to Assist with Care Decisions in Children with Autism Spectrum Disorder (ASD)

**DOI:** 10.1155/2018/9703101

**Published:** 2018-11-18

**Authors:** Arshia Khan, Kun Li, Janna Madden

**Affiliations:** Department of Computer Science, University of Minnesota, Duluth, MN, USA

## Abstract

MyHeifer is an autism spectrum disorder (ASD) intervention application aimed at better understanding patients' behavioral patterns and informing healthcare decisions, easing caregiver burden, and providing an emotional outlet for patients. Children with ASD often struggle with the complexity of human communication because of the array of verbal and nonverbal communication methods at play. Because of this, technological interventions can be a valuable tool for communicating with children with ASD because of their simplicity. Hence the MyHeifer application seeks to provide an uncomplicated environment for children with ASD to express and explore their emotions. Children perform “actions” or “interactions” which are classified as either positive or negative behaviors. Through these interactions, children learn various ways to react to situations. The choices children make are collected and serve as a basis for future healthcare decisions. Because communication is often difficult for children with ASD, utilizing data from past actions or interactions helps caregivers anticipate and understand the challenges to make better emotional and behavioral connections in individual patients in order to address personalized care needs.

## 1. Introduction

Mental health is becoming a prevalent component of the ever changing healthcare environment and a growing portion of healthcare costs worldwide. The rising demand for ASD care is, as would be expected, comparable to this trend. Since 2000, the prevalence of ASD has increased from 6.7 per 1,000 (approximately 1 in every 150 children) to 14.6 which is equivalent to about 1 in every 68 children [1]. In addition, children with ASD typically have medical expenses that exceed those without ASD by $4,110 to $6,200 annually [2, 3]. In addition, there are many indirect costs incurred by the family of patients with ASD including reduced work time due to the need to provide care for the child, increased nonmedical costs, and special educational and support needs [4]. Besides the economic factors, nationally, there is also a shortage of 70,000 providers in mental health and inequitable distribution of the providers with a greater shortage in rural areas [5]. This often requires families to wait long periods of time between appointments, travel long distances, or forgo necessary treatments because of the access limitations. In addition, primary physicians are often less prepared to treat mental health conditions [5]. While this is often where a patient is diagnosed, primary physicians often lack the resources and treatment environment to provide the necessary care for mental health conditions including ASD. In rural areas, the long distance between the patient and doctors exacerbate the difficulties of the care. The imbalance and inequity of healthcare resource allocation also restricts patients' access to the care they desire. Doctors face the difficulties of keeping up-to-date with patients' current situation. The gap between the two sides influences therapeutic effects. Because of this, patients often see many healthcare providers. The challenge of maintaining the care continuum between multiple providers is often a challenge undertaken by caregiving family members, adding to their existing stress levels [5].

Under the traditional therapy method, doctors and counselors design remedies based on the behavior records of children with ASD and professional experience. The more doctors and counselors can track the daily behavior of children with ASD, the more accurate and efficient the therapy could prove. It is impossible to expect doctors to be with children suffering from ASD at all times to monitor their stimulation responses in order to address the child's behavior and make therapy decisions [5, 6]. This discusses an application that is built to monitor and gather information on the daily behavior of a child with ASD as the first-hand record to fill the gap of data needed to design traditional therapy methods. To keep children with ASD interested and guarantee the data's steadiness and integrity, the application is supported by the back-end service, which constantly uploads new functions to maintain children's attention. It has been verified through many experiments and surveys that children with ASD are more comfortable playing with auto machines and video games. Nevertheless, the robots and games designed specifically for children with ASD are rare. The robots and games developed for the children with ASD are often set with fixed settings and cannot be personalized to a single patient's personal condition. The goal of developing the MyHeifer mobile application is to address some of the limitations and challenges facing children with ASD and their families. MyHeifer creates a virtual environment in which children with ASD can interact with an animated heifer. These interactions can be either positive or negative and serve not only as a safe outlet for patient's emotions but also as a “journal” of interactions for caregivers and healthcare providers. These empirical data can then be used to inform further healthcare decisions with the overall goal of improving the care provided to the patient. The application enables the children with ASD to select the functions where the back-end supports by adding the child's role play in order to personalize the game. This way, the application builds different play roles unique to a child. The differences between play roles reflect patient's personal mental condition to help doctors make better decisions. Several realizations have lead to the development of MyHeifer. First and foremost, caregivers are being asked to fill a gap in the patient's care. Caregivers are not only expected to support the patient on a daily basis, caregivers are also expected to monitor, evaluate, and coordinate patient care between various providers. In addition, the shortages and uneven distribution of medical professionals make getting access to the necessary health services more challenging. Through the use of MyHeifer, it is hoped that some of these locality challenges can be addressed through the use of remote patient monitoring technologies. Finally, the growing prevalence, longevity, challenging characteristics, and the gap in care continuum that was noted earlier were all factors that lead to the identification of need.

## 2. Background

ASD is a complex condition which affects brain development and has a prevalence of approximately 14.6 per 1,000 children or 1 in every 68 children [1]. ASD causes the individual to have varying degrees of difficulty handing social interactions and interaction pressure or stress surrounding verbal and nonverbal communication as well as dependency upon routines and repetitive behaviors, intense obsession with a particular idea or topic, and difficulty managing emotional responses [4, 7]. While ASD can affect how children interact, learn, or react, intelligence is not affected. Despite this, children are often frustrated by the difficulties they experience in communication, emotion, and social interaction, which can lead individuals to exhibit aggressive behavior.

There is a growth in mobile technological solutions to address ASD [8]. Current treatment strategies for ASD focus on managing conditions and improving functioning for patients as there is currently no cure [9]. There are four main treatment branches used to manage ASD symptoms: behavioral approaches, dietary approaches, pharmaceutical therapy, and complementary and alternative medical therapies. Behavioral approaches often center around the idea of helping children learn to manage and communicate the way they are feeling. One widely accepted behavioral approach is applied behavioral analysis in which positive and negative behaviors are encouraged and discouraged, respectively, through repetitive stimuli. Children can also benefit from “floortime,” a practice that focuses on emotions, developing relationships, and dealing with responses to stimuli such as sight, sound, or smell. In addition, children with ASD can also benefit from occupational therapy, sensory integration therapy, and speech therapy. Some individuals see a positive improvement when adhering to dietary restrictions or supplements; however, there is not much evidence supporting these recommendations. Medication is also used at times to control related symptoms of ASD such as depression, seizures, or inability to focus. The final category, complementary and alternative medicine, refers to treatments outside the realm of typical medicine used to alleviate symptoms; however, many of these techniques lack scientific backing [4].

Among all the treatment methods suggested, behavioral approaches to symptom management are the most widely utilized and supported methods of managing ASD in children [10]. With that being said, there is significant challenge for parents of children with ASD to provide this near-constant reinforcement and structure that is suggested by these models. Not only is it a big time commitment, providing care for a child with ASD puts significant physical and emotional stress on the caregiver [11]. For these reasons among others, assistive technology has been suggested as a tool for treating the symptoms of ASD [11, 12]. Combining these two areas of research, we seek to emulate the benefits that are seen in patients utilizing traditional applied behavioral analysis methods by adapting this method to the mobile device environment to make these interventions accessible and individualized to the needs and responses of each individual patient.

MyHeifer application offers the family members an opportunity to use the app to enable positive behavior in children affected with ASD. The app tracks the children's emotional responses and behaviors and reports them to the clinicians. This information is helpful in providing decision-making data to the clinicians, thus sanctioning the clinical decision-making process. Additionally, the app will provide statistical data to study the emotional behavior and the responses of children affected ASD. This practical application opens avenues for further research into understanding the behavior of children affected with ASD.

## 3. Methods

This application seeks to provide a positive addition to the treatment regime of children with ASD. In addition to providing children with ASD a means to expressing their emotions, this tool also provides caregivers and medical professionals the ability to analyze the decisions being made by the child within the application. As will be discussed, this adds valuable insight to the patient's care decisions.

In the development of this application, three important stakeholder groups were considered: children with ASD, parents, and healthcare providers. For children with ASD, human interactions can be very overwhelming. People communicate with their eye movements, facial expressions, head movement, and hand gestures. In comparison, interacting with a device involves much less interpretation of nonverbal communication, which can be difficult for children with ASD. In addition, this application provides a safe environment for children to express how they feel. The application is built on the framework of the applied behavioral analysis to provide encouragement for positive behaviors and discouragement of negative behaviors. Since applied behavioral analysis has proven effect in reducing negative behaviors, it is predicted that using this framework with this application will have a similar effect [10, 12].

In addition to meeting the patient's needs, this application is also designed to fit with the needs of caregivers. Caregivers play a very active role in assisting children with ASD. Caregivers monitor symptoms, administer medications, and play a crucial role in managing care continuity between various healthcare providers such as therapists and doctors. Since, these responsibilities are typically taken on by a family member, who in doing so, reduces their ability to work and adequately meet their own needs, many caregivers become physically and emotionally drained [12]. The significant role that caregivers play in the patient's care and daily life is one of the significant challenges and factors that indicated need for such an intervention. An important note in regards to caregivers is their already limited time. For this reason, the proposed MyHeifer application does not rely on caregiver intervention following the initial setup which would typically take about five minutes. Because of the demands on caregivers' times, it is important to note this application was designed in a way to reduce the caregiver's workload, not increase it. This application seeks to address the needs of the family, who are likely playing the role of caregiver as well. Caregivers often take on the role of managing care continuity and monitoring patient symptoms on top of providing daily care. To address this, the MyHeifer application saves data about the patient's interactions that are shared with providers. This reduces the demands on the caregivers.

Finally, these data created from the game can be utilized by healthcare providers to describe the patient's interactions to add insight to the care decisions. These data are presented per incident so as to show association between events and behavioral outcomes. These data become an actionable part of the patient's health records. With these stakeholders in mind, the MyHeifer application was developed to address the challenges faced in providing care for children with ASD.

**Figure 1:**
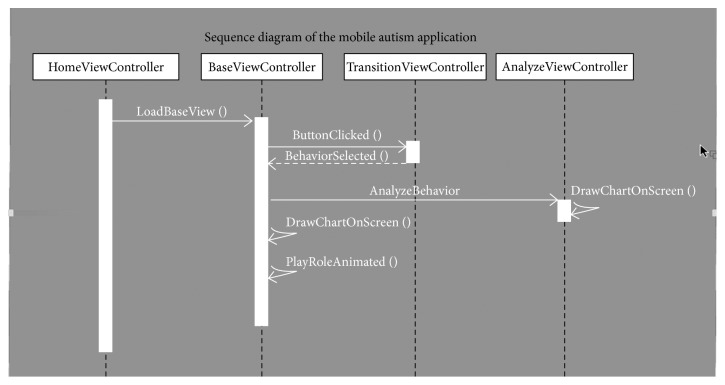
Application flow diagram.

**Figure 2:**
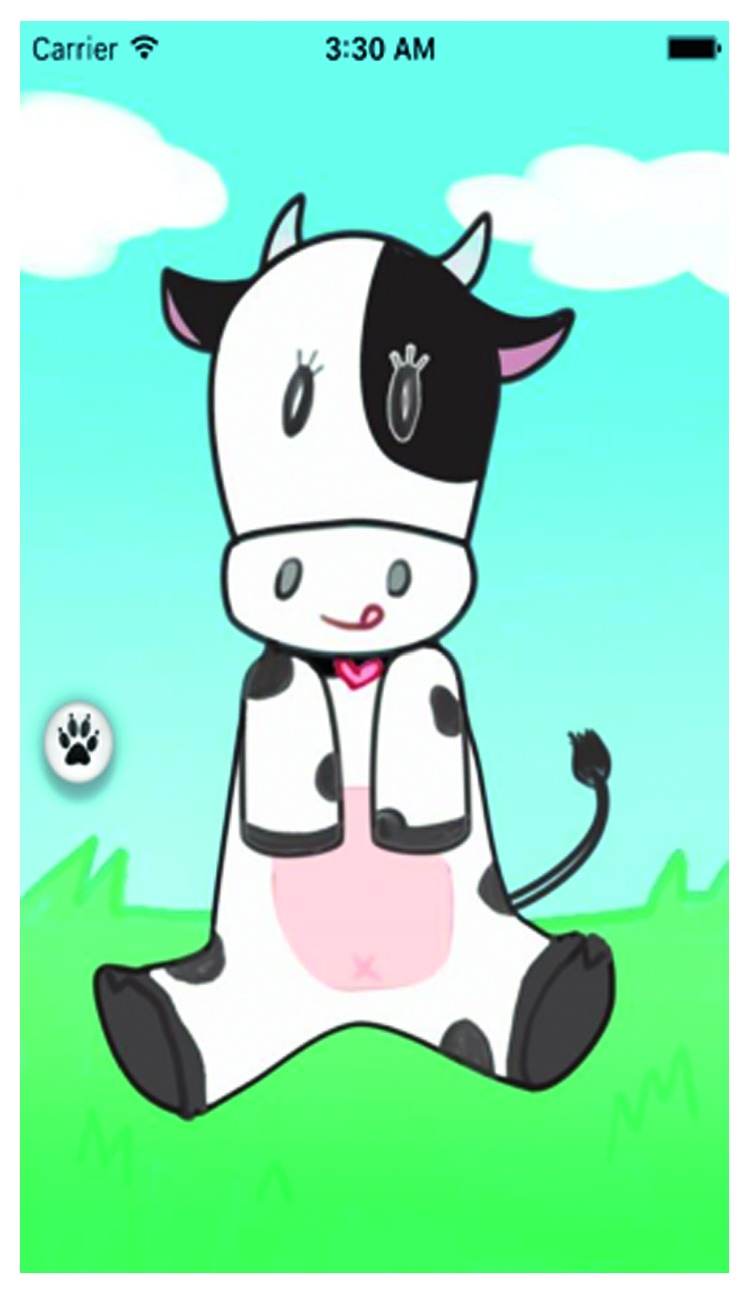
Welcome page.

**Figure 3:**
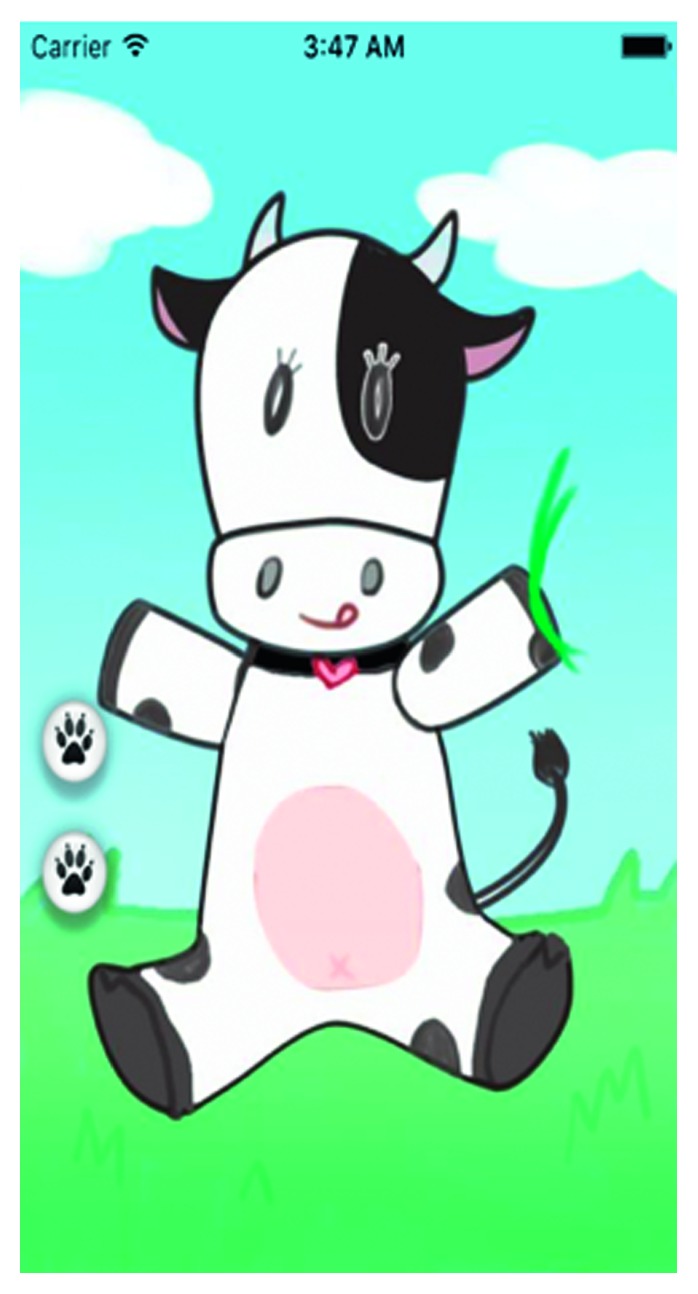
Positive action displayed.

Figure 1 shows how the application interfaces between these various roles. “HomeViewController” serves as the framework of the application. Built on top of this is the “BaseView.” This is the “starting” place of the application (or visually, the welcome page as seen in Figure 2). From this “start” position, the application transitions based on personalized decisions made by the patient. Each one of these decisions cues a “TransitionViewController” instance, which is then followed by an instance of “AnalyzeViewController.” These last two frameworks are the building blocks for the customizable and analyzable features of the MyHeifer application. “TransitionViewController” layer changes the base environment to include an additional element, for example, in Figure 3, the environment has been modified to include hay for MyHeifer to munch on. The “TransitionViewController” layer enables customization of the applications environment per the child's design. The new item calls an “AnalyzeViewController” object specifically related to it, and then proceeds to draw the new animation onto the screen and initialize the new role. It is through this initializing of the role that the visual cartoon gains personality and becomes customized to the particular patient. The proceeding interactions are now dependent upon the current role or environment being animated by the application. All of these decisions become actionable by healthcare professionals by way of the next tool: the “AnalyzeViewController” framework. The “AnalyzeViewController” is attached to each transition and saves data on each action. This enables each change to the environment to be analyzed as individual instances. This also collects data surrounding each environment being portrayed and creates data related to each instance. As will be discussed, this enables data analysis of a child's actions within the application which could enable a better understanding of a child's healthcare needs and individualized care plan requirements.

**Figure 4:**
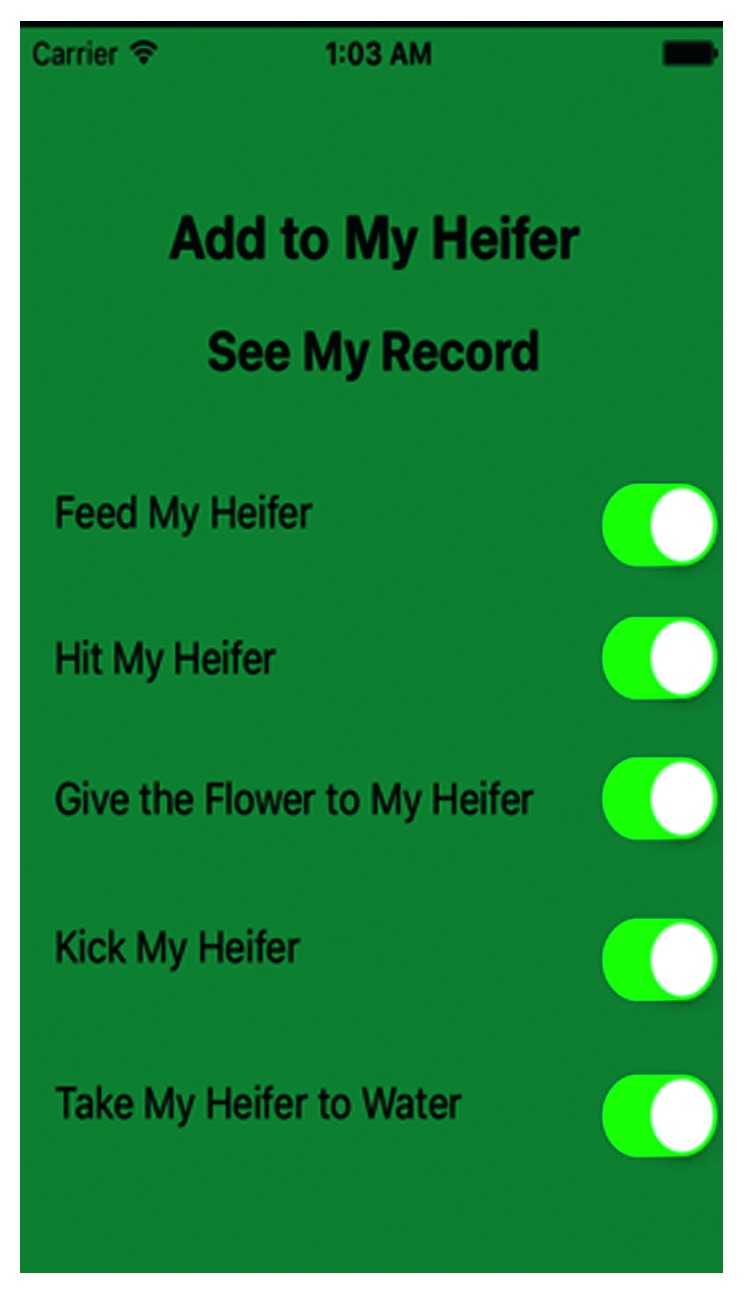
Select action.

With this basic understanding of the back-end functionality and the connection between multiple user types on the same system, it is time to consider front-end design aspects. Figures 2 and 4 show the home page and the configuration page, respectively. From the welcome page, children are able to interact with the application. They can talk to the cow and begin personalizing the cow by clicking on the “foot-print” on the left of the screen. From there, the child is directed to the menu seen in Figure 4. Here, the child can add features to their cow and personalize the play role by themselves.

**Figure 5:**
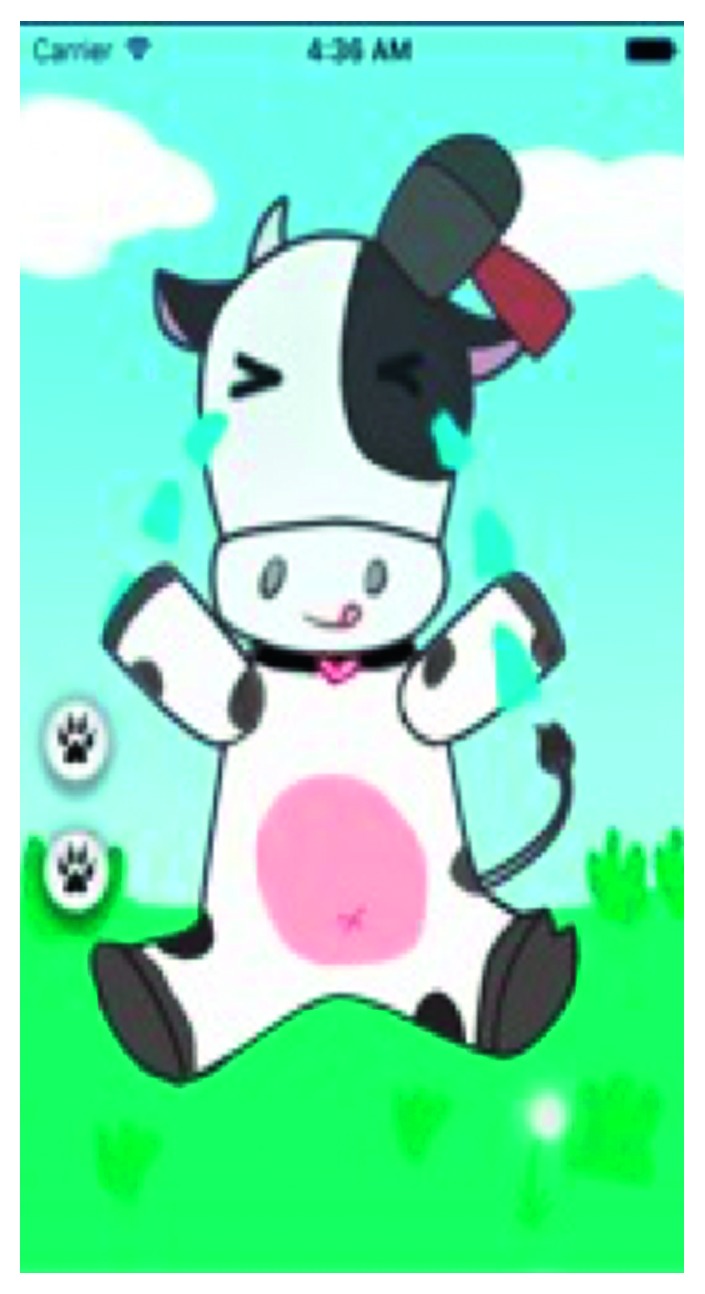
Negative action displayed.

As can be seen in Figure 4, the cow then gets these features added to graphic displayed on the welcome page. In the case of Figure 3, the cow is now holding hay in its hand. By selecting different options form the list, children are able to customize the cow while they interact with it. The cow is holding a glass of water in its hand. The cow's behavior reflects the children's emotional feelings that they tend to take care of others and be positive. By contrast, as in Figure 5, the child hits the cow with the hammer. It reflects the child tends to hurt others surrounded with them and stay negative.

The interface the child interacts with is based on a few key ideas; first, the interface is designed in a way that minimizes stressors. Unlike human interaction which is saturated with hand movements, facial expressions, and eye movement, this user interface aims to limit these behaviors which are often overwhelming. By limiting expressions and behaviors, the cow's actions are more predictable for children [12]. In addition, the application provides a way for children to express their frustrations without harming others. The application also gives more flexibility to ASD children to design their game. Since each ASD child should be offered a personalized treatment, our app is supported by individualized games. The number of positive and negative functions the children add to the play role implies their interests and mental situations. Finally, this application simulates the behavioral treatment approaches, allowing caregivers time to address their other needs and demands.

**Figure 6:**
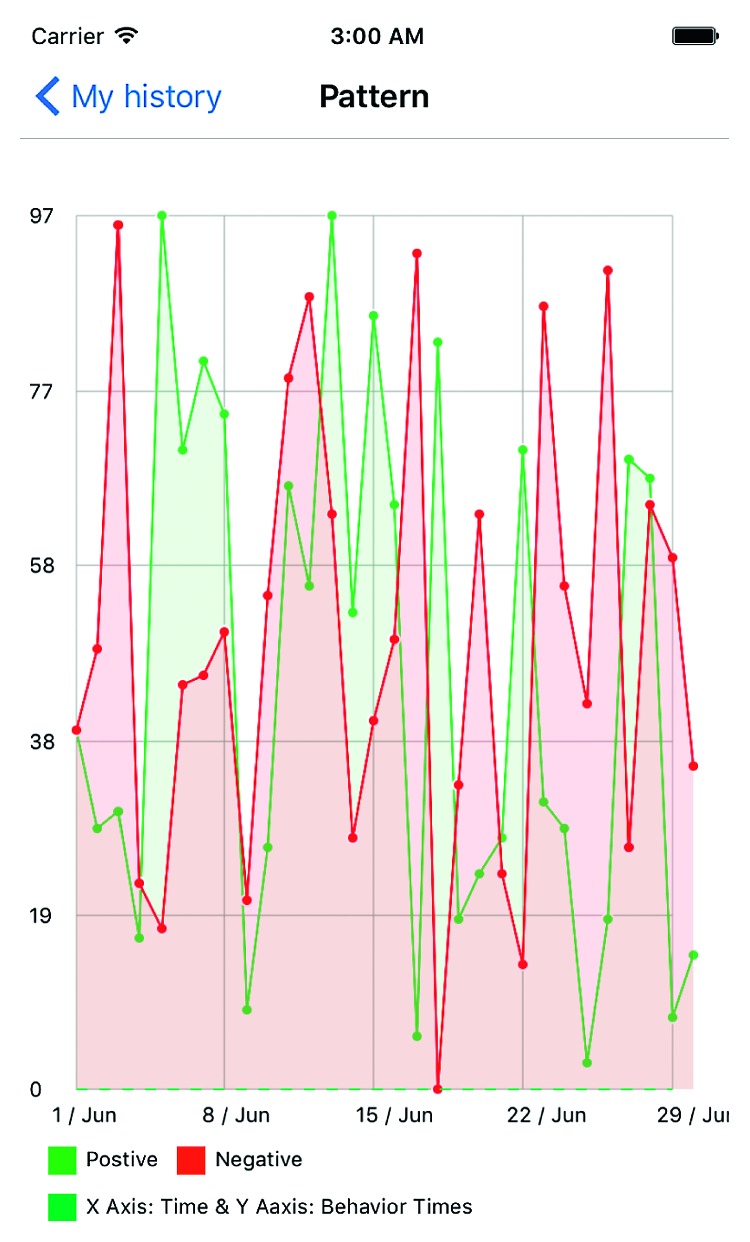
Positive versus negative decision fluctuations over one month.

**Figure 7:**
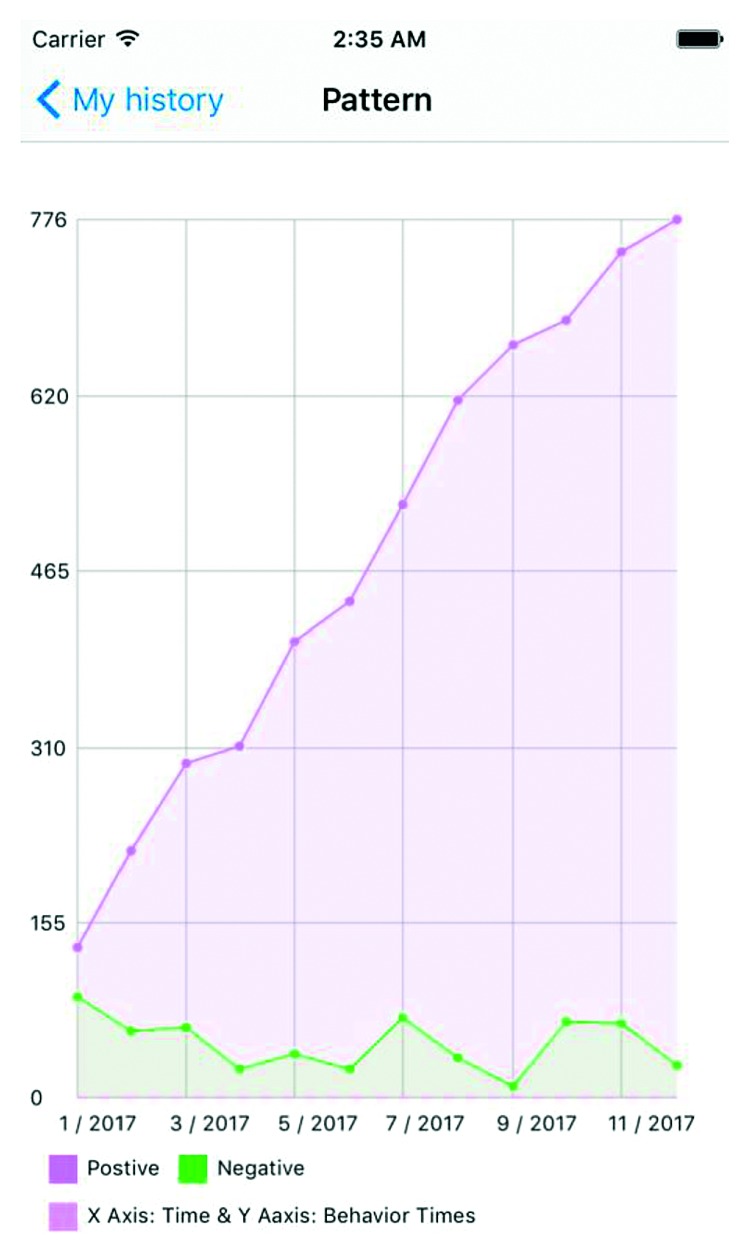
Data analysis—example one.

The data created through this application can provide information valuable for informing patient care. Besides providing caregiver with ASD children's behavior record, the application also offers built-in functions to analyze the children's emotion patterns in a specific period of time. The patient's caregiver or healthcare provider is able to analyze the data generated by the application in a variety of ways, enabling them to see what decisions the child is making within the application. This provides an idea of what emotions the child may be experiencing. For example, giving the cow food or a gift shows a positive reaction while a hurtful action would be classified as a negative reaction. The result can be seen in Figure 6. This graph shows the quantity of positive (blue) to negative (yellow) responses on a given day. Trends over a period of time can then be gathered and analyzed in order to determine future health decisions. The graph in Figure 7 achieves a similar objective of enabling analysis of the child's decisions. In addition to quantitative data, the child's conversations can be retrieved. This serves as a reference to provide the context of particularly notable series of actions or look for correlation between actions and “conversation” topics. Both the conversation record and button clicking history will be sent to the doctors as the first-hand data to improve the accuracy of therapy.

## 4. Data Interpretation

This application seeks to acknowledge the individuality of each patient with ASD. The patient's individualized data are used to benchmark the patient's trends which in turn makes the knowledge generated through the application more personalized. Children are also able to customize the character and select actions based on their own mood and emotions. This gives them a way to express their emotions which is often a challenge for children with ASD. Because caregivers and healthcare providers can analyze the child's choices, emotional patterns are more likely to be addressed by caregivers. Finally, the accumulated game history leads to improved decision making which in turn helps children better address their symptoms.

**Figure 8:**
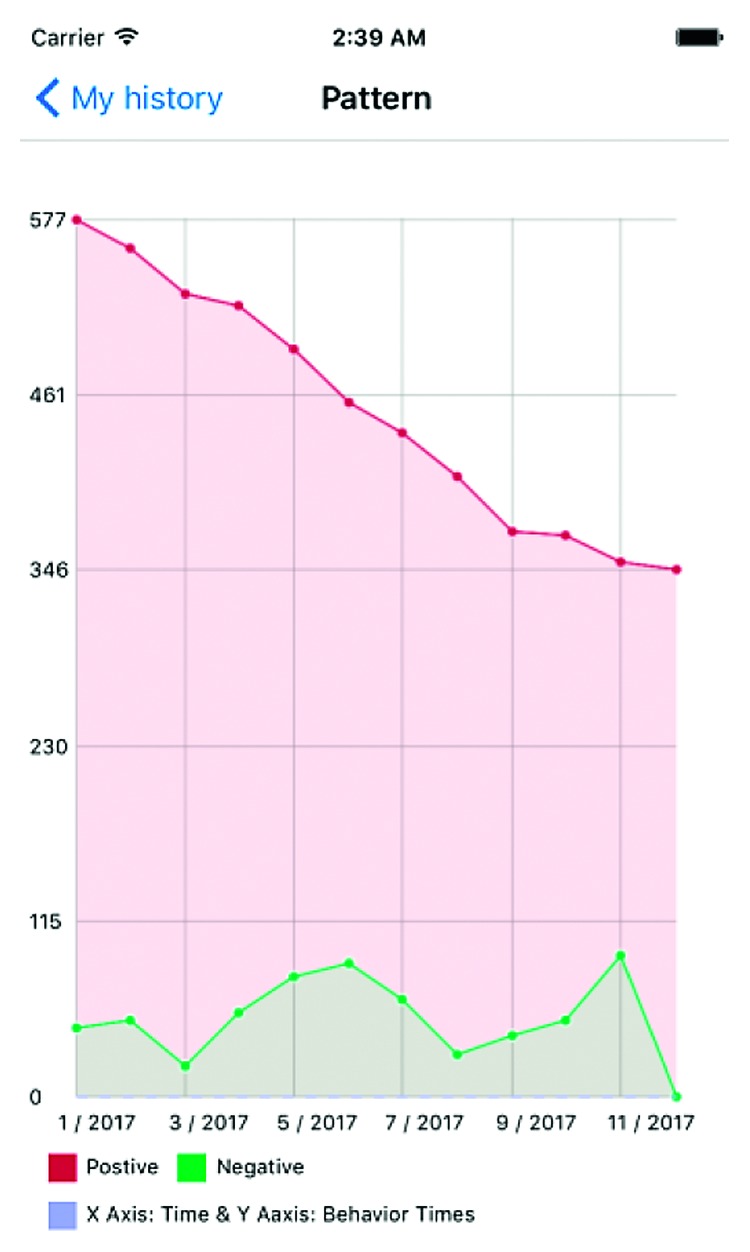
Data analysis—example two.

**Figure 9:**
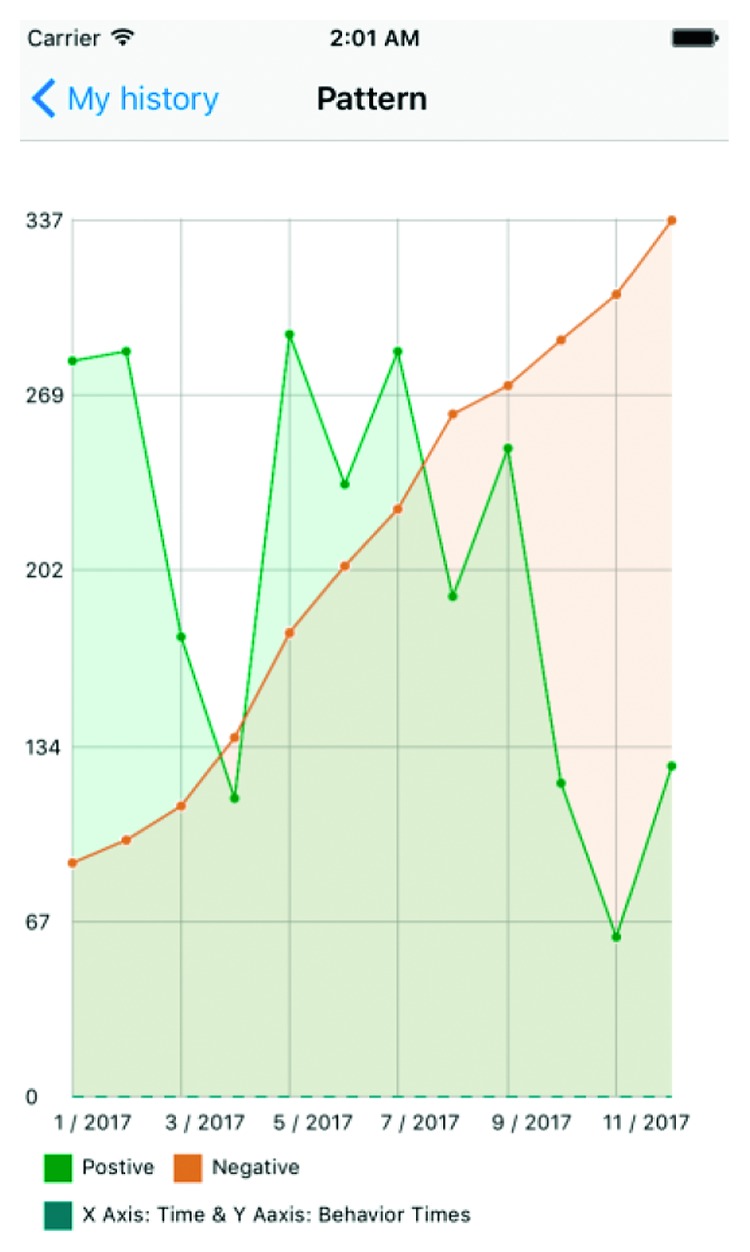
Data analysis—example three.

Figures 7–9 show an example of the type of data that would be available to healthcare providers. The *y*-axis is a count of actions (both positive and negative) over the *x*-axis of time. This gives care providers a retrospective view of the patient's positive and negative actions or choices. In Figure 7, the frequency of positive actions is steadily increasing over the time period while negative remain fairly constant and low throughout the time period. This example represents a child whose ASD symptoms are well managed and understood by the patient. The child has connected their emotions to positive behavioral outlets and has minimal negative behaviors. On the other hand, Figure 8 is mirrored after a child with ASD facing a complex emotional situation. The positive reactions are seen declining, with increased variability in negative reactions—sometimes very high, other times very low. Similarly, Figure 9 shows an increasing negative reaction rate over the time period with a variable positive reaction rate. This pattern could also indicate a child struggling with challenging social situations.

This application becomes more than a safe outlet for children's emotions when healthcare providers and caregivers are able to view data and correlate rates of positive and negative reactions with social pressures the child may be facing. One of the key benefits of this is that it provides a means to understanding children's emotional state who may not otherwise be able to communicate how they are feeling with others. In addition to being a communication tool, these data can also be used to drive healthcare decisions over a long time span by using metrics derived from this dataset. In doing so, healthcare providers could see the long-term behavioral effects of chosen interventions, and in doing so, benchmark additional interventions against each other to create a personalized care plan.

## 5. Conclusion

Just as every child with ASD faces unique challenges, so must interventions adapt to fit the individualized needs of each child. The MyHeifer application seeks to do just this by creating an environment where children can safely express their emotions and can learn how their behavioral choice impacts others. Because children with ASD often struggle with communication about feelings, this application also serves as a tool for patients and caregivers to discuss emotions and provides a tool through which patients can communicate with their care team.

To facilitate communication, this application is built upon the ideologies of many foundational ASD interventions currently being used such as floor time and applied behavioral analysis. By combining the core framework of these interventions, the MyHeifer application builds off of common interventions currently utilized in the field. The floor-time methodology is built around the idea of exposing a child to sensory stimuli and discussing emotional responses to situations. This application serves as a tool for discussing emotions and behavioral decisions. It also incorporates applied behavioral analysis in the form of behavioral choices that are categorized as either positive or negative. These behavioral choices have effects within the application environment. The combination of these functionalities makes MyHeifer an application well based in current intervention standards.

Future research seeks to look at the possibility of incorporating more data and metadata surrounding each positive or negative incident in hopes of better determining the causes of individual instances of positive or negative behavior. Through increased data analysis capabilities, it is assumed that conclusions will be more concrete and healthcare providers can feel more confident acting on these data.
